# A Clinical Data Warehouse Analysis of Risk Factors for Inpatient Falls in a Tertiary Hospital: A Case-Control Study

**DOI:** 10.1097/PTS.0000000000001163

**Published:** 2023-09-15

**Authors:** Eunok Kwon, Sun Ju Chang, Mikyung Kwon

**Affiliations:** From the ∗ Global Planning and Development Department, Seoul National University Hospital, Seoul, Republic of Korea; † Nursing Department, Sheikh Khalifa Specialty Hospital, Ras Al Khaimah, United Arab Emirates; ‡ Nursing College, Seoul National University; § Department of Pediatric Nursing, Seoul National University Hospital, Seoul, Republic of Korea.

**Keywords:** accidental fall, risk factor, electronic medical record, clinical data warehouse, case-control study

## Abstract

**Objectives:**

The aims of the study are to identify fall risk factors and to establish automatic risk assessments based on clinical data from electronic medical records of hospitalized patients.

**Methods:**

In this retrospective case-control study, we reviewed the electronic medical records of 1454 patients (292 and 1162 patients in the fall and nonfall groups, respectively) who were hospitalized at a 1800-bed tertiary hospital in South Korea between January 1, 2017, and December 31, 2017. Patients’ age, sex, and clinical department were matched, and all laboratory reports, clinical flow sheets, and nursing initial assessment records of case from the Clinical Data Warehouse system were analyzed. The collated patient records data were analyzed using SAS (version 9.4) and logistic regression.

**Results:**

Overall, 65 risk factors, including low body mass index, low blood pressure, low albumin levels, high fasting blood sugar level, low red blood cell counts, and high potassium levels, that significantly increased the incidence of falls were identified. Falls were also associated with 21 items from the clinical flow sheet and nursing initial assessment, including frequent bowel movements, 24-hour urine tests, imaging tests, biopsy, pain, intravenous tubes, unclear consciousness, and taking medication.

**Conclusions:**

Fall risk factors identified via the Clinical Data Warehouse can be used to build an automated detection system to detect fall risk in electronic medical records, enabling nurses to assess the fall risk in addition to using the fall scale.

Falls are accidents wherein a part of the body inadvertently falls or descends to a lower position than its current position. According to the World Health Organization fact sheet reported in 2021, an estimated 684,000 fatal falls occur annually, making it the second leading cause of unintentional injury-related death after road traffic injuries. In addition, more than 80% of fall-related fatalities occur in low- and middle-income countries, with regions of the Western Pacific and South East Asia accounting for 60% of these deaths. Globally, mortality rates are the highest among adults older than 60 years.

Although not fatal, approximately 37.3 million severe falls requiring medical attention occur yearly.^1^ In the “Statistics of Korea Patient Safety Reporting & Learning System (KOPS),” 27,798 cases (45.8% of 60,567 cases) of safety accidents from July 2016 to July 2022 involved falls.^2^ Falls can cause varying degrees of damage ranging from scratches to death and can result in economic losses, such as additional medical expenses, extended hospital stays, and lawsuits due to medical negligence.^3,4^

Although falls can occur in any context or setting, hospital falls have been frequently reported and may have severe consequences for inpatients. After examining the electronic medical records of 1216 fall patients, Yoon et al^5^ reported that night shift hours, hospital rooms, department of internal medicine, and age of older than 71 years were risk factors for falls.^6^ The additional risk factors of falls in previous studies were reported as disorientation, frequency of urination, gait restriction, loss of endurance, and amount of medication administered by the caregiver within 72 hours before falling.^7,8^ Factors vary according to the individual patient’s environment, personnel, and hospital culture; therefore, understanding these factors helps in decision making regarding patient safety.^9^ In a previous study of fall risk factors in older patients admitted to a general hospital, including 360 fall and nonfall patients each, fall experience and gait disturbance were considered significant using the Johns Hopkins fall risk assessment tool (JHH).^10^

Therefore, prevention of hospital falls requires multifaceted evaluation of risk factors, customized intervention, and efforts by the entire medical institution, including nurses who play a key role in managing patients.^8,11^ Fall risk assessment tools are the earliest and most important strategies for fall prevention.^12–14^ The Morse Fall Scale, St Thomas’s Risk Assessment Tool in Falling Early Infants, and Hendrich II Fall Risk Model are the most reliable tools for predicting the risk of falling in patients.^15,16^ Although nurses regularly assess falls using internationally verified fall assessment tools, fall accidents continue to occur in clinical areas. Once a patient’s clinical data becomes unstable, the laboratory results are reported, or a nurse records the observations in a clinical flow sheet and nursing initial assessment record. Immediately fall risk factors are discovered, nurses automatically raise awareness of the risks of falls from patients’ electronic medical records. Therefore, it would also be particularly useful for nurses to provide fall care without assessing the risk of falls.

Risk factor analyses of various diseases and the proposal of an automated fall risk prediction model to prevent safety accidents (such as falls) have been reported with the initiation of the Clinical Data Warehouse (CDW). Particularly, it was stated that the predictive validity after the clinical application of the completed automated detection system of fall risk would help reduce the burdens on nurses.^17–19^

Therefore, this study aimed to identify risk factors for inpatient falls using the CDW analysis.

## METHODS

### Study Setting and Data Collection

In this retrospective case-control study, we explored fall risk factors by analyzing the electronic medical records of fall and nonfall patients in 2017. Data were collected from June 1, 2018 to November 30, 2018, after obtaining approval from the institutional review board of Seoul National University Hospital (SNUH) (IRB no.: H-1804-154-942). The CDW system used in the hospital where this study was conducted was the SNUH Patient Research Environment (SUPREME). Based on the hospital research system, the fall and nonfall groups’ medical records (laboratory reports, clinical flow sheet, and nursing initial assessment) were retrospectively investigated to explore fall risk factors.

### Patient Categories

This study’s participants were patients 15 years or older who were admitted to the hospital between January 1, 2017, and December 31, 2017; they were categorized into fall and control groups. Overall, 292 patients in the fall group were reported to the quality and patient safety team, which is the patient safety management system of the hospital, due to falls during hospitalization. The control group included 1168 patients (a multiple of 4) by pairing department, age, and sex with the fall group. The 1:4 selection ratio of the fall and control groups was selected based on a previous study.^19^

Patients’ data were obtained using the hospital’s 2.0 version of the CDW—which enabled advanced search scenarios, time series data search, basic visualization of patient distribution, privacy through the IRB system, and sharing of a web-based platform in 2018. User rights were automatically granted access to full-time doctors and nursing managers of hospitals for advanced clinical research. The collected data included all items of laboratory results, including blood tests and imaging, and those of the clinical flow sheet and nursing initial assessment, such as general characteristics and patient information.

### In-Hospital Research Search System: Clinical Data Warehouse

The medical records of patients in the fall and control groups were collected through the CDW system, including data regarding fall risk factors determined using the laboratory reports, clinical flow sheet, and nursing initial assessment. When using the CDW to establish the control group, age was selected based on the birth year. If less than 4 participants were selected, the range was expanded around the birth year, whereas the department of primary diagnosis was selected if a patient’s department was changed. In addition, if the control group contained many patients, those with complete data were selected, including hospitalization date, discharge date, and diagnosis. After analyzing the distribution of fall dates in the fall group, 179 of 292 cases were confirmed to have occurred 1 to 2 days before discharge. Finally, the laboratory results and clinical flow sheets were extracted from 2 days before discharge, while the nursing initial assessment on admission was collected at hospitalization.

### Statistical Analysis

Data were analyzed using SAS version 9.4 (SAS Institute, Inc, Cary, NC). All extracted data were classified into continuous, descriptive, and categorical types. Among the extracted data, continuous variables are presented as mean and standard deviation (e.g., blood pressure, pulse, hemoglobin value, among others), whereas data of variables having a descriptive formula (e.g., computerized tomography [CT] and electrocardiography) are presented as frequency and percentage. In addition, the preexisting category was used when the result was expressed as a categorical variable in the electronic medical record (e.g., body mass index [BMI] and communication level). Therefore, to extensively explore factors affecting the occurrence of falls, this study primarily included analysis with logistic regression using univariate analysis.

### Ethical Considerations

The bioethics review committee of Seoul National University Hospital in the Republic of Korea (IRB no. H-1804-154-942) approved this study, which focused on developing a fall risk prediction tool for inpatients. In addition, the requirement for informed consent was waived because of the study’s retrospective nature. All researchers accessed the data after receiving training on research ethics. The CDW system in the hospital can be accessed by one research director and co-researchers with IRB approval to extract data. Specifically, personal information identifying the patients was excluded from the extracted data, and only data necessary for analysis were obtained.

## RESULTS

### General Characteristics of Participants

Table 1 shows the general characteristics of the participants. The average age of patients in the fall and control groups was 64.4 ± 1 and 64.1 ± 2 years, respectively, whereas 54% of the cohort were male. Among 292 falls, 171 (58.7%) and 73 (25.0%) falls occurred in the internal medicine department and hemato-oncology, respectively, demonstrating the highest incidence rates. This was followed by 33 (11.3%), 26 (23.9%), and 16 (5.5%) falls in the gastroenterology, general surgery, and neurosurgery department, respectively.

**TABLE 1:** General and Clinical Characteristics of Study Participants (N = 1460)

Characteristics	Fall Group (n = 292)		Control Group (n = 1168)	
	Mean ± SD or n (%)			
Age, y	64.40 ± 14.90		64.10 ± 15.20	
Sex				
F	134	(45.9)	536	(45.9)
M	158	(54.1)	632	(54.1)
Department (primary diagnosis)				
Internal medicine	171	(58.7)	684	(58.7)
Gastroenterology	33	(11.3)	132	(11.3)
Infectious diseases	2	(0.7)	8	(0.7)
Internal medicine (hospitalist)	16	(5.5)	64	(5.5)
Endocrinology	1	(0.3)	4	(0.3)
Cardiology	14	(4.8)	56	(4.8)
Nephrology	18	(6.2)	72	(6.2)
Hemato-oncology	73	(25.0)	292	(25.0)
Pulmonary	14	(4.8)	56	(4.8)
Surgery	70	(23.9)	272	(23.8)
Plastic and reconstructive	1	(0.3)	4	(0.3)
Neurosurgery	16	(5.5)	64	(5.5)
General surgery	26	(8.9)	104	(8.9)
Breast cancer center	3	(1.0)	12	(1.0)
Orthopedic	11	(3.8)	44	(3.8)
Thoracic and cardiovascular	13	(4.5)	44	(3.8)
Urology	4	(1.4)	16	(1.4)
Obstetrics and gynecology	10	(3.4)	40	(3.4)
Neurology	15	(5.1)	60	(5.1)
Emergency medicine	10	(3.4)	48	(4.1)
Otorhinolaryngology	5	(1.7)	20	(1.7)
Rehabilitation medicine	2	(0.7)	8	(0.7)
Neuropsychiatry	5	(1.7)	20	(1.7)

### Risk Factors for Falls

#### Laboratory Reports

Overall, 65 laboratory results were statistically significant in this study. Table 2 shows the significant variables among the tests where the laboratory results were presented as continuous variables. As the microalbumin/creatinine ratio in urine increased by 1, the fall occurrence rate decreased by 2.635 times. In contrast, the fall occurrence rate decreased by 1.903 times as the potassium concentration in the blood increased by 1. In addition, the levels of basophils (odds ratio [OR], 1.398; 95% confidence interval [CI], 1.030–1.898), fasting blood sugar (OR, 1.386; 95% CI, 1.042–1.844), phosphorus (OR, 1.299; 95% CI, 1.144–1.476), and calcium (OR, 1.261; 95% CI, 1.022–1.556) were statistically significant. As the red blood cell count and albumin level increased by 1, the fall occurrence rates decreased by 0.681 and 0.752, respectively. Many patients with severe oncology were hospitalized, and urinalysis was conducted 24 hours daily before chemotherapy. This outcome seemed to have occurred because the patients had abnormal levels of potassium or creatinine related to kidney function. Furthermore, the results of the electrolyte level, which is highly related to the high fall, seem to have been affected by the high rates of falls among oncology patients.

**TABLE 2:** Results of the Univariate Analysis: Laboratory Findings (Continuous Variables)

Items	Fall Group	Control Group	95% CI			
	Mean ± SD		LL	UL	OR	*P*
ANC	6522.24 ± 7374.60	5280.04 ± 5080.36	1.01	1.06	1.03	0.003
Albumin	3.37 ± 0.75	3.49 ± 0.61	0.61	0.92	0.75	0.006
Alkaline phosphatase	137.32 ± 133.63	100.77 ± 92.70	1.02	1.04	1.03	<0.001
BUN	26.38 ± 21.03	19.92 ± 16.31	1.01	1.03	1.02	<0.001
Basophil	0.63 ± 0.51	0.57 ± 0.39	1.03	1.90	1.40	0.032
Bilirubin, total	1.97 ± 4.69	1.06 ± 2.22	1.04	1.13	1.09	<0.001
CO_2_, total (serum)	24.01 ± 5.13	24.84 ± 3.91	0.93	0.99	0.96	0.010
Calcium	8.73 ± 0.70	8.63 ± 0.63	1.02	1.56	1.26	0.031
Eosinophil	2.27 ± 2.33	2.93 ± 2.73	0.84	0.95	0.89	<0.001
FBS (serum)	139.64 ± 58.51	98.64 ± 15.76	1.04	1.84	1.39	0.025
Fibrinogen	388.25 ± 131.75	362.44 ± 117.73	1.00	1.03	1.02	0.012
Folate	8.95 ± 5.27	6.04 ± 2.45	1.04	1.35	1.19	0.010
Glucose	135.1 ± 59.12	120.64 ± 46.45	1.03	1.08	1.05	<0.001
Hb	10.88 ± 1.88	11.23 ± 1.97	0.85	0.98	0.91	0.010
Hct	32.89 ± 5.97	34.29 ± 5.61	0.94	0.98	0.96	<0.001
Iron saturation	38.66 ± 25.86	27.73 ± 20.50	1.00	1.04	1.02	0.033
MPV	10 ± 1.44	10.38 ± 1.03	0.660	0.84	0.75	<0.001
Metamyelocyte	1.95 ± 2.20	2.89 ± 4.35	0.803	0.10	0.90	0.046
Microalbumin/creatinine ratio	1.97 ± 1.28	0.24 ± 0.60	1.21	5.76	2.64	0.015
O_2_CT	12.61 ± 3.59	13.68 ± 4.13	0.86	1.00	0.93	0.051
O_2_SAT (VBGA)	74.01 ± 16.32	58.39 ± 18.27	1.00	1.10	1.05	0.038
PCT	0.2 ± 0.10	0.23 ± 0.11	0.01	0.22	0.05	<0.001
PDW	12.53 ± 3.07	11.71 ± 2.34	1.07	1.18	1.13	<0.001
PT, %	85.99 ± 27.41	90.81 ± 22.75	0.87	0.98	0.92	0.012
PT, s	14.5 ± 7.67	13.1 ± 4.38	1.01	1.07	1.04	0.003
Phosphorus	3.7 ± 1.28	3.41 ± 0.90	1.14	1.48	1.30	<0.001
Potassium	4.35 ± 0.67	4.14 ± 0.50	1.50	2.42	1.90	<0.001
Protein (BF except CSF)	2.25 ± 1.39	3.3 ± 2.25	0.51	0.98	0.70	0.036
RBC	3.53 ± 0.69	3.7 ± 0.67	0.56	0.83	0.68	<0.001
RDW	15.94 ± 3.16	14.42 ± 2.50	1.15	1.26	1.20	<0.001
T3	60.46 ± 18.81	76.05 ± 16.66	0.91	0.10	0.95	0.028
Triglyceride	134.96 ± 68.39	98.93 ± 43.58	1.05	1.20	1.12	0.002
Uric acid	5.45 ± 2.47	4.77 ± 2.03	1.08	1.22	1.15	<0.001
WBC	135.15 ± 229.34	679.56 ± 975.34	0.70	0.96	0.82	0.015
eGFR	80.84 ± 50.52	89.52 ± 47.61	0.93	0.99	0.96	0.009
hs-CRP quantitation	4.3 ± 6.64	3.45 ± 4.80	1.00	1.05	1.03	0.031
pco_2_	37.09 ± 9.93	42.89 ± 10.92	0.91	0.98	0.94	0.001
po_2_	107.89 ± 57.08	91.08 ± 44.67	1.008	1.13	1.07	0.027
po_2_ (VBGA)	47.87 ± 14.36	36.66 ± 9.79	0.999	1.17	1.08	0.052

The significant variables are displayed in this table.

ANC, absolute neutrophil count; BUN, serum urea nitrogen; eGFR, estimated glomerular filtration rate; FBS, fasting blood sugar; Hb, hemoglobin; Hct, hematocrit; hs-CRP quantitation, high sensitivity C-reactive protein quantitation; LL, lower limit; MPV, mean platelet volume; O_2_CT, oxygen content; O_2_SAT (VBGA), oxygen saturation (venous blood gas analysis); PCT, procalcitonine; PDW, platelet distribution width; pco_2_, partial pressure of carbon dioxide; po_2_, partial pressure of oxygen; po_2_ (VBGA), partial pressure of oxygen (venous blood gas analysis); Protein (BF except CSF), protein (body fluid except cerebrospinal fluid); PT, prothrombin time; RBC, red blood cell count; RDW, red cell distribution width; T3, triiodothyronine; UL, upper limit; WBC, white blood cell count.

As shown in Table 3, patients who requested potassium, protein, and creatinine levels exam through the 24-hour urinalysis had increased risk of falls by 12.85, 30.56, and 31.38 times, respectively. In addition, significant differences were found between the genital (OR, 14.12; 95% CI, 10.00–19.93) and respiratory (OR, 9.52; 95% CI, 6.74–13.43) specific cultures and the abdominal CT (OR, 9.47; 95% CI, 6.82–13.16) and blood culture (OR, 9.15; 95% CI, 6.73–12.44) results.

**TABLE 3:** Results of the Univariate Analysis: Laboratory Findings (Binary Variables)

Items	Fall Group		Control Group		95% CI			
	Yes	No	Yes	No	LL	UL	OR	*P*
	n (%)							
HBs Ag	67 (22.9)	225 (77.1)	96 (8.2)	1072 (91.8)	2.36	4.69	3.33	<0.001
Bacteria	137 (46.9)	155 (53.1)	95 (8.1)	1073 (91.9)	7.31	13.63	9.98	<0.001
Anti-HCV	55 (18.8)	237 (81.2)	90 (7.7)	1078 (92.3)	1.93	4.00	2.78	<0.001
Creatinine (24-h urine)	289 (99.0)	3 (1.0)	881 (75.4)	287 (24.6)	9.98	98.64	31.38	<0.001
Echocardiography	74 (25.3)	218 (74.7)	87 (7.4)	1081 (92.6)	3.00	5.94	4.22	<0.001
RPR (auto)	35 (12.0)	257 (88.0)	86 (7.4)	1082 (92.6)	1.13	2.60	1.71	0.011
Protein (24-h urine)	288 (98.6)	4 (1.4)	820 (70.2)	348 (29.8)	11.30	82.62	30.56	<0.001
CT (abdomen)	117 (40.1)	175 (59.9)	77 (6.6)	1091 (93.4)	6.82	13.16	9.47	<0.001
Respiratory specimen culture	106 (36.3)	186 (63.7)	66 (5.7)	1102 (94.3)	6.74	13.43	9.52	<0.001
Genitourinary specimen culture	129 (44.2)	163 (55.8)	62 (5.3)	1106 (94.7)	10.00	19.93	14.12	<0.001
X-ray (chest)	259 (88.7)	33 (11.3)	605 (51.8)	563 (48.2)	5.00	10.68	7.30	<0.001
Casts (urine)	126 (43.2)	166 (56.8)	57 (4.9)	1111 (95.1)	10.40	21.05	14.79	<0.001
Potassium (24-h urine)	116 (39.7)	176 (60.3)	57 (4.9)	1111 (95.1)	9.01	18.32	12.85	<0.001
Protein C/S	42 (14.4)	250 (85.6)	53 (4.5)	1115 (95.5)	2.30	5.42	3.53	<0.001
Blood culture	230 (78.8)	62 (21.2)	337 (28.9)	831 (71.1)	6.73	12.44	9.15	<0.001
Bilirubin	216 (74.0)	76 (26.0)	285 (24.4)	883 (75.6)	6.57	11.81	8.81	<0.001
Color	216 (74.0)	76 (26.0)	285 (24.4)	883 (75.6)	6.57	11.81	8.81	<0.001
Glucose	216 (74.0)	76 (26.0)	285 (24.4)	883 (75.6)	6.57	11.81	8.81	<0.001
Ketone	216 (74.0)	76 (26.0)	285 (24.4)	883 (75.6)	6.57	11.81	8.81	<0.001
Nitrite	216 (74.0)	76 (26.0)	285 (24.4)	883 (75.6)	6.57	11.81	8.81	<0.001
Turbidity	216 (74.0)	76 (26.0)	285 (24.4)	883 (75.6)	6.57	11.81	8.81	<0.001
Urobilinogen	216 (74.0)	76 (26.0)	285 (24.4)	883 (75.6)	6.57	11.81	8.81	<0.001
Albumin	216 (74.0)	76 (26.0)	284 (24.3)	884 (75.7)	6.60	11.87	8.85	<0.001
Squamous cell	215 (73.6)	77 (26.4)	282 (24.1)	886 (75.9)	6.55	11.76	8.77	<0.001
X-ray abdomen	164 (56.2)	128 (43.8)	141 (12.1)	1027 (87.9)	6.98	12.48	9.33	<0.001

The significant variables are displayed in this table.

Anti-HCV, antibody to hepatitis C virus; HBs Ag, hepatitis B surface antigen; LL, lower limit; RPR (auto), auto rapid plasma regain; UL, upper limit.

#### Clinical Flow Sheet and Nursing Initial Assessment

In total, 21 nursing items of the clinical flow sheet and inpatient nursing initial assessment significantly affected falls. Table 4 shows that as the pain intensity and the number of bowel movements per day increased by 1, the fall occurrence decreased by 1.321 times and increased by 1.44 times, respectively. It was confirmed that when the diastolic and systolic blood pressures increased by 10 mm Hg fold, the rate of falls decreased by 0.802 and 0.896 times, respectively. However, the fall rate of patients with intravenous lines was 124.03 times higher than that of those without intravenous injection lines. Furthermore, patients with pain had a rate of falls that was 11.10 times higher than that of those without pain.

**TABLE 4:** Results of the Univariate Analysis: Flow Sheet and Nursing Record on Admission

Items	Fall Group		Control Group		95% CI			
	Mean ± SD				LL	UL	OR	*P*
Continuous variables								
BST	141.00 ± 58.80		182.20 ± 87.60		0.89	0.96	0.92	<0.001
Pulse rate	83.40 ± 20.20		79.80 ± 19.50		1.02	1.18	1.10	0.010
No. defecations/day	1.90 ± 2.60		1.40 ± 1.30		1.05	1.25	1.14	0.003
SBP	116.90 ± 27.60		124.90 ± 25.70		0.85	0.94	0.90	<0.001
DBP	68.90 ± 16.30		74.70 ± 15.50		0.74	0.87	0.80	<0.001
Pain intensity	2.60 ± 2.30		1.50 ± 2.00		1.07	1.63	1.32	0.010
Binary variables	Yes	No	Yes	No	LL	UL	OR	*P*
	n (%)							
Presence of IV site	266 (91.1)	26 (8.9)	89 (7.6)	1079 (92.4)	78.53	195.90	124.03	<0.001
Pain	211 (72.3)	81 (27.7)	222 (19.0)	946 (81.0)	8.27	14.91	11.10	<0.001

The significant variables are displayed in this table.

BST, blood sugar test; DBP, diastolic blood pressure; IV, intravenous injection; LL, lower limit; SBP, systolic blood pressure; UL, upper limit.

As demonstrated in Table 5, patients were 6.00 times more likely to fall when underweight (BMI, 18.5 kg/m^2^) than when mildly obese (BMI, 25–30 kg/m^2^), and they were 3.22 times more likely to fall when overweight (BMI, 23–25 kg/m^2^). However, patients with an orientation to time had a 0.36 times lower rate of falls than those without the orientation to time. The patients who received recent medication showed a rate of falls 1.94 times higher than those who did not. Furthermore, in the initial assessment of hospitalization, the rate of falls was 1.20 times higher in patients with pain than in those without pain. Moreover, the patients who could not move freely had 2.47 times higher rates of falls than those who could move freely.

**TABLE 5:** Results of the Univariate Analysis: Nursing Record on Admission (Categorical Variables)

Items	Fall Group	Control Group	95% CI			
	n (%)		OR	LL	UL	*P*
BMI						<0.001
<18.50	32 (20.9)	70 (9.3)	6.00	3.11	11.58	
18.50 ≤ BMI < 23.00	56 (36.6)	263 (34.9)	2.79	1.56	5.01	
23.00 ≤ BMI <25.00	43 (28.1)	175 (23.2)	3.22	1.76	5.92	
25.00 ≤ BMI <30.00	16 (10.5)	210 (27.9)	Ref.			
≥30.00	6 (3.9)	36 (4.8)	2.19	0.80	5.96	
Previous admission history						0.003
No	237 (28.7)	27 (16.0)	Ref.			
Yes	586 (70.9)	142 (84.0)	2.13	1.37	3.30	
Previous disease history						
No	3 (0.4)	0 (0.0)	—	—	—	<0.001
Yes	276 (33.4)	29 (15.6)	Ref.			
Not evaluated	545 (66.0)	157 (84.4)	2.74	1.80	4.18	
Orientation to time						0.0340
No	9 (5.8)	17 (2.2)	Ref.			
Yes	140 (90.9)	734 (95.8)	0.36	0.16	0.83	
Not evaluated	5 (3.2)	15 (2.0)	0.63	0.17	2.30	
Response to pain						0.0390
No	4 (2.6)	5 (0.7)	Ref.			
Yes	147 (97.4)	74.2 (99.3)	0.25	0.07	0.93	
Body weight change						0.0120
No	137 (82.5)	722 (89.5)	Ref.			
Yes	29 (17.5)	85 (10.5)	1.80	1.14	2.85	
Recent medication						0.0370
No	12 (7.1)	106 (12.8)	Ref.			
Yes	158 (92.9)	720 (87.2)	1.94	1.04	3.61	
Pain						0.030
No	106 (56.4)	517 (62.6)	Ref.			
Yes	72 (37.3)	292 (35.4)	1.20	0.86	1.68	
Not evaluated	10 (5.3)	17 (2.1)	2.87	1.28	6.44	
Movement						<0.001
Free to move	115 (76.2)	663 (88.8)	Ref.			
Not free to move	36 (23.9)	36 (23.8)	2.47	1.60	3.83	

The significant variables are displayed in this table.

LL, lower limit; UL, upper limit.

## DISCUSSION

This study’s results demonstrate that falls were most common in the internal medicine department, particularly hemato-oncology. Overall, 65 risk factors significantly increased the fall occurrence rate, identified using univariate analysis. In addition, 21 items were statistically significant in the clinical flow sheet and nursing initial assessment. The highest fall occurrence rate was in patients of the internal medicine department, which aligns with the results of several previous studies.^6,19^

Internal medicine includes patients with many chronic diseases, such as cardiovascular disease, malignant tumors, and digestive system disease. Patients are usually accompanied by complications and various diseases with symptoms including general weakness, dizziness, and excretion and are frequently prescribed various fall-related drugs. Among internal medicine, the largest number of patients in hemato-oncology and the highest level of neurosurgery in surgery are consistent with the highest frequency of fall occurrences in previous studies, which were associated with nausea, vomiting, dizziness, or decreased visual function due to chemotherapy complications.^19^

Among the many variables, the most significant risk factor for falls was the presence of an intravenous injection site in patients. Particularly, patients with an intravenous injection site had a fall rate 124.03 times higher than those without an intravenous injection site. This result aligns with that of Choi et al,^20^ who reported that 63.3% of fall patients had an intravenous peripheral vein. The presence of intravenous injection sites acts as a risk factor for falls because the patients’ conditions are severe and require an intravenous injection site for medication or nutritional fluid due to insufficient diet. In addition, it is believed that falls may occur because of collision with patients’ feet and the intravenous injection pole stand’s feet while moving.

Although several other variables were found in this study, a poor medical condition was also confirmed as a risk factor for falls. This study’s results indicate that patients who requested a biopsy, imaging, urine, and blood culture tests had a higher rate of falls than those without any requests. Patients’ conditions were still complex despite the various evaluation studies conducted for patients 2 days before discharge.

People with walking difficulty or who are unconscious are also at high risk of falling. Choi et al^20^ showed that individuals with weak gait or those who cannot move without the assistance of surrounding people or devices were 1.37 and 1.62 times more likely to fall than others, respectively. Among the results that can quantify a patient’s medical condition, we found that a lower albumin level, red blood cell count, and BMI indicated a higher incidence of falls. The weakening of lower limb muscles correlates with fall risk, indicating that lower BMI, rather than obesity, may affect fall occurrence. Therefore, comprehensively evaluating nutritional status when determining the risk of falling in patients is necessary in the future. According to Kim et al,^21^ the risk of falling is 2.075 times higher if physical weakness is present; albumin level and red blood cell count in this study can be considered quantifiable test results.

Fall occurrence was higher in patients who underwent a 24-hour urinalysis, microalbumin test, and test for potassium and phosphorus levels. Because 24-hour urinalyses are frequently performed to confirm renal functions in patients with cancer before administering chemotherapy, fall occurrence is commonly associated with oncology patients. The microalbumin/creatinine ratio has significance in predicting diabetic and hypertensive kidney disease. Specifically, creatinine, protein, and potassium (24-hour urinalysis) levels are also used to evaluate kidney function, suggesting the associations of these items with underlying diseases.

Regarding pain, patients with pain during hospitalization and those complaining of pain during hospitalization had an extremely high risk of falling. Kim and Choi^22^ reported that patients with pain were 1.68 times more likely to fall than those without pain. In this study, the risk of falls increased with pain intensity; therefore, the risk of falls should be lowered through pain management and assessment of the presence and intensity of pain. Furthermore, patients taking medication had a higher risk of falls than those not taking medication; however, further analysis is required because this was not analyzed according to the type of medication and the number of doses.

Lee et al^19^ reported that a high pulse was associated with falls. Although pulse was not associated with falls in this study, we observed that lower blood pressure led to a higher rate of falls. Blood pressure is a variable representing the state of body fluid, and low blood pressure is highly associated with falls. In a study by Kim et al,^22^ patients with dizziness reported a higher risk of falls at 6.205 times. Fall occurrence was also higher in this study with the presence of hypotension, low red blood cell count, and low hemoglobin level.^23^

Our study attempted to extract fall risk factors using patients’ data stored in electronic medical records. Although using large-scale data of inpatients in the data collection process are advantageous, there is a limit to the comprehensive extraction of drugs related to the risk factors for falls and age because the fall group was selected via matching by the medical department, age, and sex. Therefore, more accurately identifying the risk factors for falls by analyzing additional records, such as medication records, is necessary in the future.

## CONCLUSIONS

The study demonstrated that falls were associated with low BMI, low blood pressure, low albumin levels, high fasting blood sugar, low red blood cell counts, high potassium and phosphorus levels, and frequent bowel movements. Patients who underwent 24-hour urinalysis and imaging tests (including CT and biopsy) and those with pain had a higher rate of fall occurrence. In addition, more falls occurred in patients with intravenous tubes, difficulty walking, and unclear consciousness, as well as in those taking medication.

In this study, the validity of the analysis was increased by conducting a patient control study where the comparable control group numbers were extracted by four times that of the fall group. Large-scale data stored in the hospital research search system were used. In the data collection process, the fall and control groups were selected via matching by the medical department, age, and sex. Because only the test results, clinical observation records, and nursing information survey sites at hospitalization were analyzed, comprehensive identification of falls-related drugs is limited. Therefore, accurately identifying the risk factors for falls through repeated studies is necessary in the future. Furthermore, if an automated risk prediction system for falls is developed that reflects these research results in the future, it will not only reduce the work of nurses who directly perform fall evaluations but also contribute to patient safety.
